# Tuberculosis Immunoreactivity Surveillance in Malawi (Timasamala)—A protocol for a cross-sectional *Mycobacterium tuberculosis* immunoreactivity survey in Blantyre, Malawi

**DOI:** 10.1371/journal.pone.0291215

**Published:** 2024-05-24

**Authors:** Hannah M. Rickman, Mphatso D. Phiri, Helena R. A. Feasey, Hannah Mbale, Marriott Nliwasa, Robina Semphere, George Chagaluka, Katherine Fielding, Henry C. Mwandumba, Katherine C. Horton, Emily S. Nightingale, Marc Y. R. Henrion, Kuzani Mbendera, James A. Mpunga, Elizabeth L. Corbett, Peter MacPherson

**Affiliations:** 1 Clinical Research Department, London School of Hygiene & Tropical Medicine, London, United Kingdom; 2 Malawi Liverpool Wellcome Programme, Blantyre, Malawi; 3 Department of Clinical Sciences, Liverpool School of Tropical Medicine, Liverpool, United Kingdom; 4 Helse Nord TB Initiative, Kamuzu University of Health Sciences, Blantyre, Malawi; 5 School of Health & Wellbeing, University of Glasgow, Glasgow, United Kingdom; 6 Department of Paediatrics, Queen Elizabeth Central Hospital, Blantyre, Malawi; 7 Department of Infectious Disease Epidemiology, London School of Hygiene & Tropical Medicine, London, United Kingdom; 8 Malawi National Tuberculosis and Leprosy Control Programme, Lilongwe, Malawi; 8th Medical Center of Chinese PLA General Hospital, CHINA

## Abstract

*Tuberculosis (TB) transmission and prevalence are dynamic over time*, *and heterogeneous within populations*. *Public health programmes therefore require up-to-date*, *accurate epidemiological data to appropriately allocate resources*, *target interventions*, *and track progress towards End TB goals*. *Current methods of TB surveillance often rely on case notifications*, *which are biased by access to healthcare*, *and TB disease prevalence surveys*, *which are highly resource-intensive*, *requiring many tens of thousands of people to be tested to identify high-risk groups or capture trends*. *Surveys of “latent TB infection”*, *or immunoreactivity to Mycobacterium tuberculosis (Mtb)*, *using tests such as interferon-gamma release assays (IGRAs) could provide a way to identify TB transmission hotspots*, *supplementing information from disease notifications*, *and with greater spatial and temporal resolution than is possible to achieve in disease prevalence surveys*. *This cross-sectional survey will investigate the prevalence of Mtb immunoreactivity amongst young children*, *adolescents and adults in Blantyre*, *Malawi*, *a high HIV-prevalence city in southern Africa*. *Through this study we will* estimate the annual risk of TB infection (ARTI) in Blantyre and explore individual- and area-level risk factors for infection, as well as investigating geospatial heterogeneity of Mtb infection (and its determinants), and comparing these to the distribution of TB disease case-notifications. We will also evaluate novel diagnostics for Mtb infection (QIAreach QFT) and sampling methodologies (convenience sampling in healthcare settings and community sampling based on satellite imagery), which may increase the feasibility of measuring Mtb infection at large scale. The overall aim is to provide high-resolution epidemiological data and provide new insights into methodologies which may be used by TB programmes globally.

## Background

*Tuberculosis (TB) causes over 1*.*5 million deaths per year*, with recent trends of falling global incidence set back by the COVID-19 pandemic [1]. *As TB incidence declines, transmission is becoming increasingly concentrated in spatial hotspots and among vulnerable populations, especially within cities [2–4]. Public health campaigns require real-time, sub-district surveillance to identify high-risk groups and geographical areas in order to allocate resources and track progress over time [5].*

*Existing methods of TB surveillance, predominantly based on TB disease rather than infection, do not meet these needs [6]. Diagnostic algorithms for TB disease may include* symptom screening (insensitive and non-specific [7]), and diagnostic tests which are often expensive, challenging to operationalise, and may lack specificity (chest radiography) and/or sensitivity (microbiological tests of sputum). *TB disease notifications are often the only routine data available to national TB programmes*. *However*, *they require a person with TB to be diagnosed and registered by the health system* [8,9]; but only 60% of people estimated to have developed TB globally in 2021 were notified [1]. Furthermore, differential access to healthcare may result in case notifications systematically underestimating disease burden in the most underserved and vulnerable populations [8,9]. Cross-sectional disease prevalence surveys have been promoted as a way of more accurately measuring TB burden and overcoming the challenge of under-notification [7]. However, with existing tools, prevalence surveys are extremely resource-intensive and imprecise at subnational levels, especially as prevalence falls. Thus, it is currently impractical to achieve district, or subdistrict-level spatial resolution, identify high-risk populations, or measure trends over time through repeated disease prevalence surveys [6].

Surveys of immunoreactivity to *Mycobacterium tuberculosis* (Mtb) or “latent TB infection” (LTBI), rather than TB disease, are an alternative way to monitor TB epidemiology [6]. Mtb immunoreactivity is defined by a positive T-cell-mediated immune response to Mtb antigen, measured using tests such as interferon gamma release assays (IGRAs) or tuberculin skin tests (TSTs), without any clinical or microbiological evidence of active TB disease [10]. Infection/immunoreactivity surveys capture an earlier stage in TB pathophysiology prior to progression from infection to disease, and so may provide clearer insights into which groups are most exposed to TB transmission as well as providing an earlier indicator of changing TB epidemiology in a population. Infection prevalence is higher than disease prevalence, since only a minority of Mtb infections result in disease, providing greater power and precision from a smaller sample size [6]. Finally, in populations at high risk of progression from infection to disease (such as young children, and people living with HIV) there may be individual-level benefits to diagnosis and early treatment of Mtb infection [11,12].

Tuberculin surveys, for example amongst primary schoolchildren or military recruits, were widely used in the last century to monitor TB epidemiology [13,14], despite the logistical challenges of requiring two contacts with providers, and placement and interpretation by trained professionals. Both the sensitivity and specificity of TSTs are sub-optimal, particularly in populations with a high prevalence of immunosuppression, *Bacillus Calmette*-*Guérin* (BCG) vaccination, and/or non-tuberculous mycobacterial (NTM) exposure [13,15]. IGRAs–*in vitro* blood tests that measure the immune response to Mtb-specific antigens not present in BCG or most NTMs–overcome many of these limitations [16]. Newer Mtb-specific tests, such as the QIAreach QuantiFERON-TB (QIAreach QFT) (which requires only a single 1mL blood sample and can be operated in a self-contained battery-powered device) [17], and specific skin tests [18], offer opportunities to expand access to TB immunoreactivity testing. TB immunoreactivity surveys will be more sustainable if they deploy easy-to-use diagnostic tests, are integrated within existing healthcare systems, and are acceptable to communities.

Blantyre City, Malawi, exemplifies many of the current challenges and epidemiological knowledge gaps facing TB Programmes in the Global South. Blantyre is a densely-populated, resource-limited urban setting with a population of 800,000 and an estimated adult HIV prevalence of 14% [19]. A 2019–2020 TB prevalence survey found a greater than 80% decline in the prevalence of undiagnosed microbiologically-confirmed TB over six years compared to the 2013–2014 Malawi National TB Survey [20], although the impact of COVID-19 is yet to be fully appreciated [21]. The 2019–2020 survey tested over 15,000 participants to identify 29 individuals with TB disease, demonstrating that even large-scale, resource-intensive prevalence surveys may be of limited value in generating granular epidemiological data. Furthermore, despite widespread public health efforts, the prevalence-to-notification ratio in Blantyre is estimated at 4.5, suggesting considerable undernotification [22]. High variation in case-notification rates has been observed between neighbourhoods of Blantyre City [9] with a pattern consistent with an “inverse care law”, where case notification rates are lower in poorer neighbourhoods with poorer access to healthcare [9]. The risk is that targeting limited resources towards areas with higher case notifications may simply divert them away from already underserved communities with a high burden of undiagnosed disease. Therefore, while there is an urgent need to accurately determine the distribution of TB disease risk within cities like Blantyre, disease prevalence surveys are now prohibitively resource-intensive and case notification rates provide at best biased estimates of burden, limiting utility for health planners, TB programmes and researchers.

Little is known about the risk factors for Mtb infection in Malawi. Globally, risk factors for TB *disease*, such as male sex, alcohol, diabetes, HIV, smoking, and malnutrition are well-described [1]. They represent a composite of the risk of infection and the risk of progression; it is less clear which groups and populations are specifically at highest risk of Mtb *infection*. Isolating risk factors for infection adds information about how and where transmission is taking place, and helps to predict the potential public health impact of interventions such as targeted preventive therapy or vaccinations amongst different groups. It is expected that these risk factors act at the individual level, at the household level, and at the neighbourhood level, and that they may differ by age.

Our aim is to understand the spatial and population variation in Mtb infection risk in Blantyre, Malawi, through a cross-sectional survey of Mtb immunoreactivity in children aged 1–5 years, and in adults and adolescents aged 10–40 years. We will explore the feasibility of different survey methodologies, including convenience sampling of children aged 1–5 years in Primary Health Clinics (PHCs) compared to community-based household sampling and, in a nested diagnostic accuracy evaluation, assess a novel diagnostic (QIAreach QFT) that may expand access to large-scale population-based screening. We aim to provide high-resolution epidemiological information about this urban area, which will be of value to policymakers locally, alongside insights into methodologies which may be used by TB programmes globally.

## Methods

This is a cross-sectional epidemiological survey, investigating the population-level prevalence of Mtb immunoreactivity amongst young children, adolescents and adults in Blantyre, Malawi. The specific objectives are to:

1. Determine the annual risk of TB infection (ARTI) in Blantyre, and explore the individual- and area-level risk factors for Mtb infection.
2. Explore the geospatial distribution of Mtb infection in Blantyre, and test the hypothesis that areas with high TB case notifications have higher rates of Mtb infection.
3. Evaluate the performance of QIAreach QFT relative to QuantiFERON-TB Gold Plus (QFT-Plus) in population-based samples of healthy children, adolescents and adults.
4. Compare the outputs of convenience sampling in Primary Health Clinics with that obtained from conventional cross-sectional community sampling, with respect to the estimated ARTI and the characteristics of participants recruited.
5. Evaluate the feasibility and acceptability of performing surveillance using blood tests for Mtb infection and other infectious diseases.

### Setting

The Timasamala (TB Immunoreactivity for Surveillance in Malawi; Timasamala is Chichewa for “We care”) study will be conducted in Blantyre, Malawi. An enhanced surveillance collaboration between the Malawi-Liverpool-Wellcome Programme, Blantyre District Health Office, and the National TB and Leprosy Programme has prospectively recorded the clinical, microbiological, and demographic data of all individuals with notified TB since 2011. Since 2015 it has additionally recorded their home locations using a validated electronic participant location (ePal) application based on high-resolution satellite images and locally-relevant geotagged points of interest [9,23]. Recruitment will commence in late 2022 and conclude by June 2024.

### Study population

**Fig 1:**
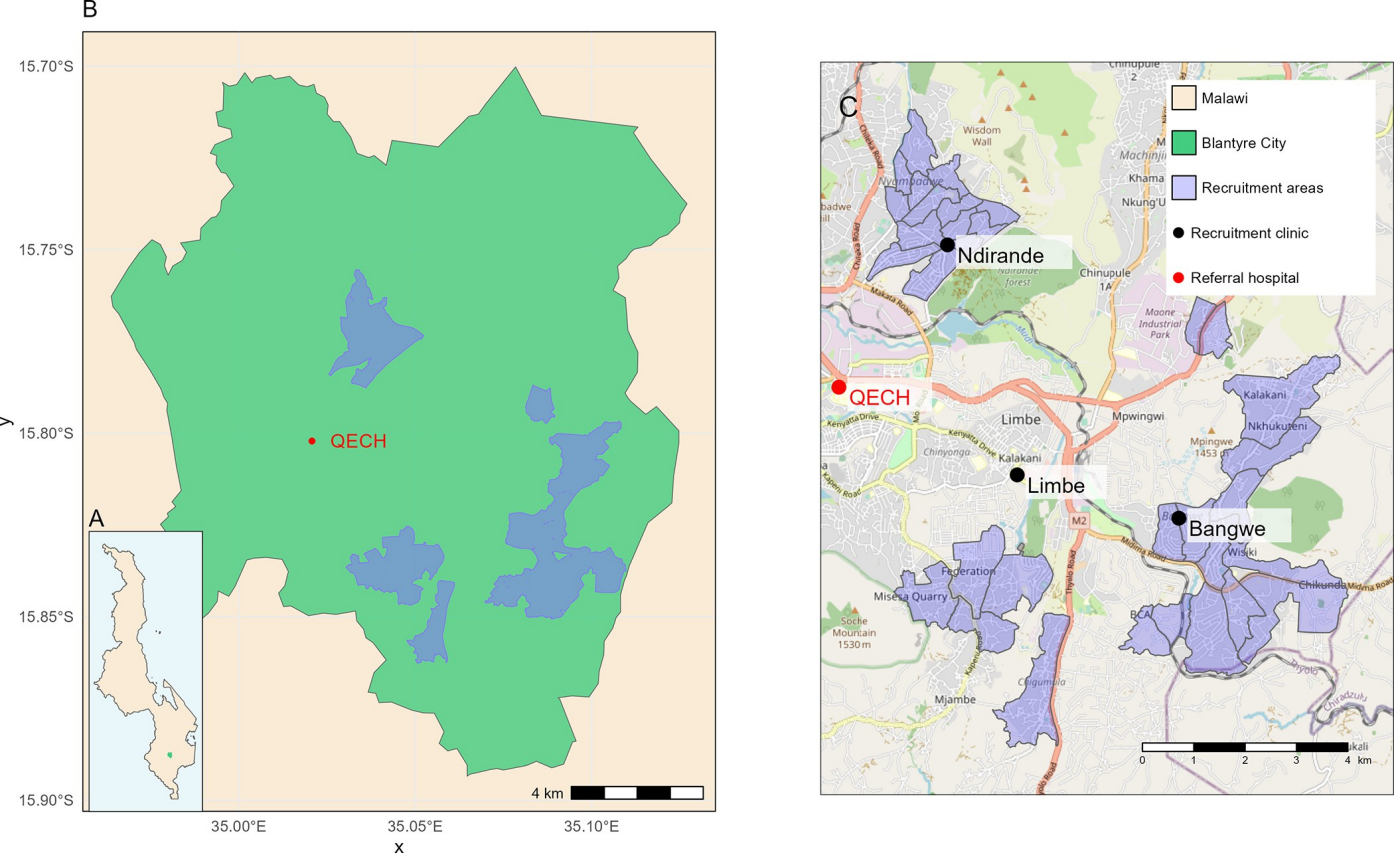
Map of Timasamala recruitment sites. A. Location of Blantyre City within Malawi. B. Location of Timasamala recruitment sites within Blantyre City. C. Detailed map of clinic and community recruitment sites. QECH: Queen Elizabeth Central Hospital. Map tile data from OpenStreetMap (https://www.openstreetmap.org/copyright). Malawi boundary data from the United Nations Office for the Coordination of Humanitarian Affairs, Humanitarian Data Exchange (https://data.humdata.org/dataset/geoboundaries-admin-boundaries-for-malawi).

Recruitment will be focussed on neighbourhoods of informal urban settlements in Blantyre (Fig 1). We will recruit from two types of settings (PHCs and the community). Clinic-based recruitment will be performed in the three PHCs in Blantyre with the highest absolute number of TB case notifications (Bangwe, Limbe and Ndirande); collectively these three clinics accounted for 58% of TB diagnoses in primary care between 2016 and 2020. Community-based recruitment will be performed in neighbourhoods which encompass the approximate catchment areas of these three clinics. These 33 neighbourhoods, previously delineated for a TB prevalence survey, were defined according to Ministry of Health Community Health Worker catchment areas and geographic boundaries; each has a population of around 4,000 adult residents [20]. Clinic catchment areas were defined through the home locations of people diagnosed with TB, captured through enhanced surveillance at each clinic [9,22,23], in addition to unpublished data from healthcare-use questionnaires conducted at the Malawi Liverpool Wellcome Programme. To ensure that community and clinic samples are comparable, clinic participants will only be included if they are resident in the 33 neighbourhoods. These two sampling strategies will allow comparison of the results of Mtb infection screening in children recruited from conventional cross-sectional household sampling, with children recruited using convenience sampling of those attending local health clinics for routine primary health services. Residence in catchment areas and households will be defined as 1) participant considers the location to be their main residence, and 2) they normally live and share meals with other members of the household.

### Recruitment procedures

#### Recruitment in clinics

Children aged 1–5 years will be identified for recruitment from those attending vaccination clinics, and from those accompanying other people attending routine primary health services, such as antenatal and postnatal care, family planning, and cervical cancer screening. On each day of recruitment, children’s guardians will be approached sequentially by a trained study nurse for consideration of enrolment and, if guardians consent, children will be recruited either while waiting (with care taken that they do not lose their place in the queue) or after finishing their routine visit, until a daily recruitment target is met.

#### Community sampling

Children aged 1–5 years, and an additional cohort of adolescents and adults aged 10–40 years, will be eligible for recruitment from households in the study communities. Enumeration of possible household structures within each geographically-defined area has been performed using data from Open Buildings, a large-scale open-access dataset developed from a deep-learning model trained to identify building footprints from high-resolution satellite imagery [24]. Population weights will be defined using open-source gridded population data from WorldPop [25]. Population-weighted random samples will be drawn from the sampling frame of potential building structures within each study neighbourhood, and selected households will be visited by field teams (who will also capture the Global Positioning System (GPS) location of the dwelling, to validate the sampling approach.) Trained study team members will discuss the study with household members and recruit participants on the same visit if they consent. All eligible household residents (or their guardians) will be approached for potential recruitment, with a second visit if required to recruit additional household members not present on the first visit. Where it is not possible to recruit from a location (e.g. a building structure is not a household, or household members decline to participate) teams will visit the next sequential location from the sample until the sample size is reached.

#### Inclusion and exclusion criteria

Inclusion criteria are deliberately broad, as the aim is to recruit a community-representative population within these age groups.

*Inclusion criteria*

1. Children aged 12–60 months (over 1 and under 5 years) of age *(clinics and community)*, adolescents 10–17 years and adults 18–40 years old *(community only)*.
2. Normally resident in one of the study neighbourhoods defined above
3. In clinics: attending for a routine appointment (e.g. vaccination clinic), or accompanying a family member or other person attending for care (e.g. mothers attending for antenatal care).
4. In community: normally resident in the household.

*Exclusion criteria*

1. Participant or guardian respectively decline to consent.
2. Presenting to PHCs because of their own symptomatic illness.
3. Participant already recruited from the other recruitment group (i.e. from community if recruited in PHCs, or PHCs if recruited in community).

### Sample sizes

#### Rationale for sample sizes

There are limited data on the prevalence of TB immunoreactivity in Blantyre on which to base sample size calculations. The last National Tuberculin Survey in Malawi in 1994 (which did not include Blantyre) found a 12.6% prevalence of TST-positivity amongst children aged 6–11 years, corresponding to an ARTI of 0.6–1.4% [26], with considerable variation by region. In a 2012 tuberculin survey of children aged 2–4 years in Karonga, a rural district in northern Malawi, TST positivity prevalence was 1.1% and the estimated ARTI was 0.3% [15]. Urban areas such as Blantyre are known to have substantially higher incidence of TB disease than rural areas, and in a recent study of 165 adults attending HIV services in urban Blantyre, 44% had a positive IGRA [27]; however the incidence of TB has been falling over the past decade [20], which may result in a lower-than-expected prevalence in young children. In early pilot work, the prevalence of IGRA positivity in children under 5 years was 6/78 (7.7%, 95% CI 2.9–16.0%), with a mean age of 2.4 years, giving an estimated ARTI of 3.3%.

#### Sample sizes for children aged 1–5 years

We have based sample size estimates on an ability to precisely estimate a prevalence of 5% with an absolute precision of ±1% (i.e. 4–6%), corresponding to an ARTI of 2.1% (1.7–2.5%) with 95% confidence. This requires a sample size of 1,825 children aged 1–5 years. Of these, we would anticipate around 91 children to have a positive QFT-Plus IGRA. We therefore plan to recruit a total of 1,825 children under 5 with a QFT-Plus result, *or* 100 children with a positive QFT-Plus IGRA–whichever milestone is reached first–from each of the clinic and community recruitment groups.

#### Sample sizes for adults and adolescents

For this objective, we will test the hypothesis that TB immunoreactivity in adolescents and young adults in Blantyre City differs by sex. Based on pilot data, we have assumed an Mtb infection prevalence among females of 15% in adolescents aged 10–17, 20% and 25% in adults aged 18–25 and 26–40 years, respectively. While there are little data from Malawi to inform estimates of the expected sex differences in prevalence, results from adults attending HIV services in Blantyre found a male to female (M:F) ratio of Mtb infection prevalence of 1.6 [27], while data from other settings [28–30] show ratios of 1.2 to 1.8, which themselves vary by age. We have therefore based our sample size on an ability to detect a M:F ratio of 1.35 in each age category, with 80% power and 95% confidence. This requires a sample size of 1,652 (826 male, 826 female) in adolescents aged 10–17, and 1,150 (575 male, 575 female) and 850 (425 male, 425 female) in adults aged 18–25 and 26–40 years respectively. We have not adjusted sample sizes for clustering at household or area level. Firstly, there are no recent data from Malawi to inform clustering estimates of Mtb infection. Secondly, we found no strong basis to assume clustering of Mtb infection at household/area level, as Mtb infection in older age groups may reflect historical exposure trends, which will likely differ even among older people living in the same household or neighbourhood. In analysis (see below) however, we will explore clustering of Mtb infection at household and area level from our data. We will additionally explore implications of such clustering, if any, on potential loss of precision on estimated Mtb immunoreactivity prevalence, and male-to-female ratios.

### Study procedures

**Fig 2:**
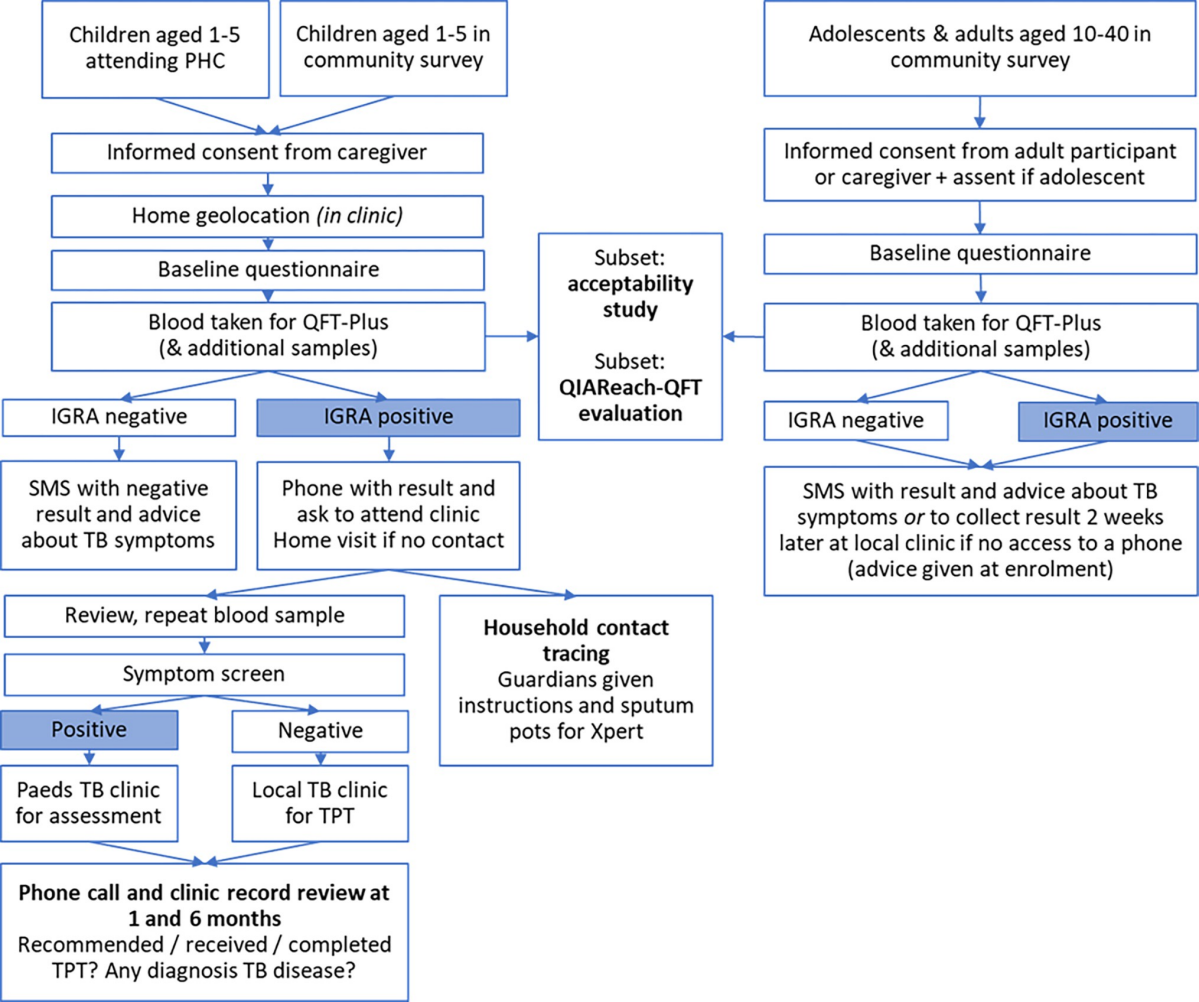
Summary of study procedures. PHC: Primary health clinic. QFT-Plus: Quantiferon-Plus. IGRA: Interferon gamma release assay. TB: Tuberculosis. SMS: Short message service. Paeds: Paediatric. TPT: TB preventive therapy. Xpert: Xpert MTB/RIF.

Study procedures are summarised in Fig 2.

### Baseline questionnaire

Participants and/or their guardians (depending on the age of the participants; adolescents may complete the questionnaire if they are able to) will be asked to complete a questionnaire which includes questions about demographics, household composition, socioeconomic status, medical history, TB contact and current symptoms. Data will be entered onto encrypted, password-protected tablets by researchers using Open Data Kit systems [31].

#### eLocate

Guardians of participants recruited in clinics will be asked to provide their (or the child’s) GPS home coordinates using the electronic participant location (ePal) application, which has previously been validated and is used to geolocate record the homes of TB patients in Blantyre [9,23]. A random 5% sample of participants will be selected for independent verification of home coordinates; study teams will visit their home (using the captured GPS coordinates and the verbal description of their address provided in the questionnaire), and compare the true GPS location with that recorded using ePal for the purposes of quality assurance.

#### QFT-Plus IGRA testing

A 4mL venous blood sample will be taken for IGRA testing using QFT-Plus, following manufacturer’s instructions. Briefly, samples will be drawn into lithium heparin tubes and transported at room temperature to the laboratory on the same day for transfer into QFT-Plus blood tubes within 12 hours of sample collection, followed by overnight (16–24 hours) incubation at 37°C. Samples will then be centrifuged, and harvested plasma tested using the QFT-Plus assay according to manufacturer’s instructions. A second venous blood sample (maximum volume 1mL) will be taken for either QIAreach QFT IGRA, additional Mtb infection assays, or serum for storage.

#### Results and follow-up

*Communication of results (children 1–5)*. Negative IGRA results will be communicated by short message service (SMS), along with standard advice about seeking healthcare if they experience any symptoms of tuberculosis. To facilitate results communication to those who may lack access to their own phone, guardians will also be given the option to attend their local clinic after 2 weeks to collect the results, and will also be given the phone number of a study representative (which they can use if they have temporary access to a phone). Guardians of children aged 1–5 will be informed that all positive results will be delivered either by phone call or, if they do not have access to a phone, via a home visit. Guardians of any children testing positive will be asked to bring their child to the PHC for an in-person review, repeat symptom screen and a follow-up blood sample by the research team, and will be given transport money to do so.

*Asymptomatic children aged 1–5 with a positive IGRA*. Asymptomatic children with a positive IGRA (defined based on standard definition using manufacturer’s instructions) will be referred to the PHC TB clinic, with a recommendation to initiate TB preventive therapy (TPT) if there are no contraindications. The World Health Organization (WHO) recommends that TB contacts under 5 years old be considered for treatment of “latent” TB infection, given their high risk of progression to active and disseminated disease. TB household contacts under 5 years of age with a positive TST/IGRA have an estimated 19% chance of developing TB over the next 2 years, and the effectiveness of preventive therapy is estimated at 91% [11]. By contrast, the risk of severe (Grade 3+) adverse events in children receiving 6 months of isoniazid (6H) (the current preventive therapy regimen recommended by the Malawi National TB and Leprosy Programme for young children exposed to TB) is estimated at 0.2% [32,33]. (While alternative TPT regimens are also recommended by WHO, these are not widely available for children in Malawi, nor included in the current Malawi National Guidelines for treatment of under-5 TB contacts.) Asymptomatic children will be contacted at 1 and 6 months, and the records of the relevant TB clinics checked to see if they have attended and received/completed TB preventive therapy, and whether they have developed active TB disease or any adverse events. Those who have not attended will be actively traced.

*Symptomatic children aged 1–5 with a positive IGRA*. If children have TB symptoms on the review visit, they will be referred to the paediatric TB clinic at the Queen Elizabeth Central Hospital (a tertiary-level referral hospital in Blantyre) to be investigated for active TB disease, and will be provided with money for transport. (All diagnostic tests are available free of charge in the government health system). They will receive a diagnostic work-up according to the standard clinical procedures (typically includes a clinical examination, blood tests, chest radiograph and stool and/or gastric aspirate samples for Xpert MTB-Rif/Ultra). Clinic staff will be asked to complete a proforma detailing any investigations performed, the results, and whether the child initiated TB treatment. Children’s guardians will be contacted at 1 and 6 months to ascertain outcomes, which will be cross-checked against clinic records.

*Adolescents and adults*. WHO does not recommend routine TB preventive therapy in adults and adolescents without HIV or other risk factors [34], and current Malawi guidance does not include provision for preventive therapy for older household contacts of people with TB. In this group, the risk of developing active TB disease in the 2 years following after a positive IGRA/TST result is estimated to be below 5% [35,36], and the risk of side effects from preventive therapy is higher than in children (estimated at 1.5% in people aged 18–34) [33]. A positive test of TB exposure does not require any specific management in this age group in the absence of other risk factors, and a negative test does not exclude the possibility of TB in the future. All participants will therefore be given advice at the time of enrolment, to seek care at a clinic if they develop TB symptoms, and to seek HIV testing if their HIV status is unknown. Those with HIV will be asked if they have previously received TB preventive therapy and, if they have not, advised to ask their ART clinic about this. Adolescents and adults testing positive will be notified of their results via SMS, with a generic reminder message of information given during recruitment about implications of a positive test result (including the need to seek care if they develop symptoms; participants with HIV will be advised to inform their ART clinic of the result). They will also be given the phone number of a study representative, and if they do not have access to a telephone will be able to attend their local clinic after 2 weeks to collect the results.

#### QIAreach QFT diagnostic accuracy study

In a nested diagnostic accuracy study, the first 1,000 sequentially-recruited participants will have a 1mL paired sample taken for QIAreach QFT, which will be taken directly into the QIAreach QFT sample tube, transported to the laboratory, incubated overnight and then analysed using the QIAreach QFT eHub and eStick (according to manufacturer’s instructions). Results will be compared against paired samples processed using the gold-standard QFT-Plus assay, and sensitivity, specificity, and Cohen’s kappa (with 95% confidence intervals) calculated for both the overall cohort, and subgroups including different age groups and people living with and without HIV. We will also compare quantitative (time to positivity) QIAreach QFT values with quantitative interferon-gamma levels from QFT-Plus.

#### Additional assays and diagnostics

Surveillance based on blood tests may be more efficient if other diseases are also included [37]. A subset of consenting participants will also have an additional saved blood sample taken (specific consent will be sought for this and participants can decline additional blood tests while still consenting to participate in the wider study). This may be processed in future for evidence of exposure to other infections, such as malaria, arboviruses, CMV or respiratory viruses, in order to explore the spatial distribution of other conditions and to evaluate the possibility of combining TB surveillance with serosurveillance for other infectious diseases, and may also be used to evaluate novel tests of TB exposure or infection, including CRP, CRISPR-TB [38], and mycobacterial serology. Participants will not be routinely informed of the results of these tests, unless they reveal clinically-relevant information about active infection. Detailed protocols for additional nested studies will be submitted for ethical approval.

#### Household contacts of children aged 1–5 with a positive IGRA

All adult household contacts of children aged 1–5 years with a positive IGRA will be advised to test for active TB disease. Guardians will be given one sputum pot per household member, with pre-filled laboratory request forms and specimen bags, and verbal and written (in Chichewa and with pictorial tools) instructions on how to safely produce a single sputum sample [39]. Guardians will be asked to return sputum pots and cards to the study nurse at the clinic from which their child was recruited, and reimbursed for their travel. All sputum samples will be tested using Xpert-MTB/Rif in government laboratories, and the results checked by the clinic nurses. The proportion of household members returning a sputum sample, and the prevalence of Xpert-positive pulmonary TB amongst household contacts, will be calculated.

#### Acceptability of Mtb infection surveillance

The participant questionnaire will include items asking about acceptability of the test. A subset of adult participants ≥18 years and guardians from both households and the clinic will be invited to a more in-depth qualitative study on these themes, with around 10 semi-structured in-depth interviews, using an interview guide, performed with guardians from each setting. Adult participants ≥18 years or guardians who decline participation in the overall research study will also be invited to participate in the qualitative study, to ensure that the views of those who did not consent to Mtb infection testing are also included. These interviews will be conducted by trained qualitative researchers, in participants’ first language. These will be supplemented by key-informant interviews with research staff and healthcare workers in the clinic.

### Data analysis

#### Prevalence of Mtb immunoreactivity and the annual risk of TB infection (ARTI) in Blantyre

The primary study outcome is the prevalence of Mtb immunoreactivity (number of children with a positive IGRA/number tested, expressed as a percentage) in Blantyre. From this, and the mean age of children tested, the mean ARTI can be calculated using[40]:

ARTI=1−(1−Prevalence)1Age

Similarly, age- and sex-specific prevalence of TB immunoreactivity and mean ARTI among adolescents and adults will be calculated.

Mtb infection tests are interpreted by converting a continuous immunological measure (IFN-γ release or skin induration) into a binary positive/negative result. There is incomplete consensus on the most appropriate thresholds [35] or whether the same thresholds are appropriate for capturing individual risk (influenced by age, HIV status and other factors) *v*.*s*. population exposure. Quantitative IGRA responses are associated with the intensity of recent TB exposure [41] and with risk of progression to active TB [42]. Sensitivity analyses will also be performed to explore the use of different quantitative cut-offs for QFT-Plus positivity, and to model immunoreactivity as a continuous variable.

#### Risk factors for Mtb immunoreactivity

Multilevel models will be developed to examine the individual-, household- and neighbourhood-level risk factors for Mtb immunoreactivity (Table 1). We will specifically explore whether a household contact with TB, and reported time spent in congregate spaces, independently increase the risk of Mtb infection in young children.

**Table 1:** Risk factors for Mtb infection, and sources of data.

Variable	Data source
**Outcome variable**	
Mtb immunoreactivity	QFT-Plus IGRA assay (binary result) Quantitative results (from assay IGRA values) will also be analysed in a sensitivity analysis
**Individual variables**	
Age	Timasamala CRF
Sex	
Self-reported HIV status	
BCG vaccination status	
Other health information	
Occupation (adults)	
Time spent in congregate spaces	
Nutritional status (under-5s)	Mid-upper arm circumference (measured in Timasamala)
**Household variables**	
Household composition	Timasamala CRF
Household TB contact	
Education level of head of household	
Household poverty level	Timasamala CRF, using validated socioeconomic index questionnaire
Distance from health services	Geolocated home location and calculated transport time to nearest health facility
**Neighbourhood variables**	
TB case notifications	Geolocation of home addresses using ePal–completed routinely by National TB Program Officers in Blantyre for all registering patients (15,000+ from 2015-present) [9,23]
TB prevalence estimates	2019–2020 SCALE disease prevalence survey of 15,897 adults [20]
TB case fatality rate (surrogate for TB care)	Dataset of registered TB patients since 2015
Population density	WorldPop estimates[25]
Poverty estimates	Timasamala household survey Household survey in TB prevalence survey (2018–2019) [20] WorldPop estimates [25]
HIV prevalence and ART coverage	Blantyre HitTB (2014) and SCALE (2019–2020) HIV prevalence surveys [20,43]

Mtb: Mycobacterium tuberculosis. IGRA: Interferon gamma release assay. CRF: Case report form. TB: Tuberculosis. ART: Antiretroviral therapy.

#### Comparison of sampling methodologies

We will investigate whether children recruited by each sampling methodology (convenience sampling in PHCs vs community-based household sampling) differ with respect to their demographic and health characteristics. We will additionally investigate whether TB immunoreactivity prevalence differs by recruitment methodology, once other factors which may differ between the two sampling strategies are controlled for (by incorporating this into a variable in the models outlined above).

#### Geospatial distribution of Mtb immunoreactivity

We will describe the continuous geospatial distribution of Mtb immunoreactivity prevalence in children under 5 across the included areas. The dependent variable is Mtb immunoreactivity (primarily modelled as a binary outcome, but sensitivity analyses may include alternative cut-offs and continuous distributions). We will capture the precise home GPS coordinates of participants and can therefore estimate a continuous distribution of Mtb immunoreactivity prevalence across the city using a Bayesian geostatistical model.

Using an unadjusted model, we will investigate whether there are spatial hotspots of Mtb immunoreactivity in Blantyre (i.e. areas in which posterior risk estimate exceedance is above a specified cut-off), and describe their spatial scale and the magnitude of the variance in prevalence.

We will explore whether these hotspots correspond to areas of high population-adjusted case notification rates, and to previously-identified areas of possible underdiagnosis. We will formally explore whether incorporating the spatial distribution of case notification rates explains some of the spatial variance of the model–i.e. do high case notifications predict high levels of TB transmission or, as we suspect, are case notifications a poor predictor of the burden of undiagnosed TB?

#### Developing spatial models of Mtb immunoreactivity to explore drivers of spatial hotspots

We will develop a geostatistical model of Mtb immunoreactivity prevalence which incorporates the individual- and household-level variables explored in the epidemiological analysis, as well as risk factors operating at a neighbourhood level e.g. population density and poverty.

Once other spatial covariables have been adjusted for, we will explore whether living close to an individual with notified tuberculosis is a risk factor for TB immunoreactivity and, if so, over what spatial and temporal scale this occurs. We will further examine for evidence of residual auto-correlation between Mtb infection prevalence, which may correspond to areas of high transmission, and which can then be compared with the results of ongoing sequencing analyses.

#### Evaluation of the performance of QIAreach QFT relative to QFT-Plus in population-based samples of healthy children, adolescents and adults

Samples for the novel QIAreach QFT IGRA will be taken for a subset of participants enrolled in the study, and run in parallel to QFT-Plus. The sensitivity, specificity, positive predictive value and negative predictive value of QIAreach QFT will be calculated against the reference standard QFT-Plus, in the whole population and in subgroups, including children under 5. Quantitative QFT-Plus and QIAReach results will also be compared.

### Feasibility and acceptability of performing surveillance using blood tests for Mtb infection and other infectious diseases

We will summarise responses to questionnaires about acceptability of testing for tuberculosis infection and compare results between different demographic groups. Qualitative interviews with participants and key informants will be transcribed and translated. Coding will be performed using NVivo (Lumivero, 2020) by at least two researchers using an inductive approach, and themes identified.

### Data management

Data management will be performed in conjunction with the Malawi-Liverpool-Wellcome (MLW) Data Department, and in accordance with Malawi, UK and EU data protection regulations and best practice guidelines, including Good Clinical Practice and Clinical Data Acquisition Standards Harmonisation (CDASH) guidelines. Paper source documents will be stored securely. Electronic data will initially be collected on encrypted, password-protected tablets using Open Data Kit systems, and then transmitted via encrypted secure internet connection to secure MLW servers. Written consent will be requested for anonymised individual data to be made openly available along with the statistical code required to reproduce analyses. De-identified datasets will be assigned a persistent URL and deposited with detailed, searchable metadata in a shared catalogue. All potentially identifiable information will be removed to ensure anonymity of shared data; for example, GPS coordinates of an individual’s residence will be replaced with an aggregated area-level location marker, and all datasets will be reviewed to ensure that a combination of demographic characteristics does not allow participants or households to be identified.

### Community engagement

The MLW TB/HIV Community Scientific Advisory Board (a panel of volunteer community members affected by TB and HIV) has provided invaluable input into the development of this protocol, including around the acceptability of testing for Mtb infection, the consent process, community recruitment strategies and communication of results. The study has been introduced to clinic staff in the three PHCs, and to the Blantyre District Health Office TB Officers, who all provided input and feedback. In the community we have presented to community leaders, local chiefs and on public radio, and sensitisation in conjunction with the MLW Science Communication Team will continue as recruitment begins. Results of the study will be disseminated to community leaders and to the Community Scientific Advisory Board.

### Ethical issues

#### Informed consent

Adults and adolescents ≥ 18 years, and guardians (Legally Acceptable Representatives; generally, a close relative of the participant who may act on their behalf) of participating children who meet inclusion criteria, will be provided with oral and written information about the study by the research team. They will be invited to provide individual informed consent, either in writing or, if illiterate, by a thumbprint confirmation which will be observed and recorded by a witness independent of the study team. Additionally, adolescents aged 10–17 years will be provided with oral and written information about the study and asked for their assent to participate. Consent forms will be stored securely. All study staff are trained in informed consent and Good Clinical Practice.

Participants will be compensated a small monetary amount for their time and transport costs, according to published local guidance [44].

#### Possible positive and negative impacts of testing for Mtb infection

Taking blood from participants, especially children, may cause distress. Trained experienced healthcare professionals will conduct all study procedures. The small volume of blood being taken is not expected to cause any health problems. General infection prevention and COVID-19 protocols will be followed to protect the safety of participants and staff.

Testing for evidence of TB immunoreactivity involves testing for a pre-disease state, which may never lead to active TB disease. A diagnosis of Mtb infection may cause distress and stigma. Great care will therefore be taken to explain the nature of Mtb infection, particularly that it is not infectious to others. The potential benefit of household members being screened for active TB disease if a participant tests positive for infection will also be highlighted. Specific questions and focus groups with participants will address the questions of understanding and acceptability of testing for Mtb infection.

WHO recommends that TB contacts under 5 years old be considered for treatment of “latent” TB infection, given their high risk of progression to active and disseminated disease [11], the high efficacy of preventive therapy [11], and the low risk of serious adverse events in this age-group [32,33].We therefore recommend that children under 5 years in this study with a positive IGRA, by definition representing recent TB exposure, are appropriately investigated for active TB and receive TB preventive therapy once active TB has been excluded. The approach to adults and adolescents with a positive test of TB infection is less clear; current Malawi guidelines do not routinely recommend TPT for adult and adolescent household contacts of people with TB; furthermore older participants in this study are not household contacts with a known, recent exposure but rather healthy community members who may have been exposed to TB years ago, who have a low risk of progression to TB disease [35,36] and an appreciable risk of side effects from preventive therapy [33]. Nevertheless as countries undergo epidemiological shifts towards TB elimination it becomes more important to understand the individual- and community-level risks and benefits of Mtb infection screening and treatment [6].

#### Approvals and regulatory issues

Ethical approval has been received from both COMREC (Kamuzu University of Health Sciences Research Ethics Committee) (Ref P.04/22/3611) and LSHTM (Ref 2774). Approval has been received from the Blantyre District Health Office to conduct the study within PHCs.

## Discussion

### Potential impact

This cross-sectional survey aims to investigate the population-level prevalence of Mtb immunoreactivity amongst young children, adolescents and adults in Blantyre, Malawi, and to produce high-quality spatial epidemiological data which can be directly used to inform policy. Despite modelling work suggesting that targeting interventions towards high-risk areas can increase efficiency and disproportionately impact transmission [45], two systematic reviews [2,3] identified just three studies describing explicitly spatially-targeted TB interventions, all from the USA [46–49]. One barrier to implementation of such targeted interventions is accurate and reliable identification of areas of high spatial risk. In addition to spatial hotspots, this study aims to identify high-risk groups and vulnerable populations who may benefit most from focussed public health efforts, and provide estimates to inform further modelling work to estimate the population-level impact of interventions targeted towards preventing Mtb infection.

While standardised methodologies have been developed for performing tuberculin surveys [50] and TB disease prevalence surveys [51], there is little consensus on some important design considerations for the conduct of IGRA surveys [6]. This study therefore aims to address unfilled knowledge gaps around the most appropriate methodologies for performing surveillance based on Mtb infection, which may inform similar surveys in other settings, and ultimately support the development of standardised methodologies to support collation by international bodies and integration with other data sources.

### Justification for methodological decisions

We have elected to recruit a large sample size of children aged 1–5 years, but also to include participants from older age groups, in response to the advantages and disadvantages of surveying different age groups for Mtb immunoreactivity [6]. Immunoreactivity in young children by definition indicates recent (i.e. within the lifetime of the child) TB exposure, and may therefore act as a useful measure of contemporary community-level transmission. Furthermore, children under 5 years of age are at high risk of progression to active and invasive TB disease, making them a priority target group for TB preventive treatment [11,34] in whom there may be individual benefit to screening for Mtb infection outside of the current recommendations for close contacts of infectious TB patients. However, transmission risks to young children do not fully reflect wider population-level epidemiology, and additional insights may also be gained from Mtb surveillance in older age groups. Adolescents and young adults are at high risk of acquiring Mtb infection [52–54], and different risk factors for Mtb infection are likely to be relevant (in particular, sex differences in TB disease emerge in these age groups [54]). Understanding Mtb infection epidemiology in these age groups allows us to better model the potential impact of preventive and treatment interventions, including different vaccination strategies. Our approach will allow us to accurately estimate contemporary ARTI in young children, while also evaluating the utility of sampling in different groups and understanding age- and sex-related dynamics of Mtb infection.

IGRA testing using QFT-Plus has several advantages over tuberculin skin testing. IGRAs use Mtb-specific antigens and are not affected by prior BCG vaccination or infection with (most) NTM; they also use a blood sample and therefore do not require a second contact with healthcare providers to read the result. However, there are significant barriers to widespread use of IGRAs in surveillance, including cost of assays, the need for a relatively high volume of whole blood (4mL for QFT-Plus), and the reliance on complex laboratory infrastructure. In a nested study we will therefore evaluate the QIAreach QFT, which has been developed to overcome some of these limitations. The QIAreach QFT requires a single 1mL blood sample, and minimal laboratory processing, using a semi-automated lateral flow immunoassay in a battery-powered device to measure IFN-g production [17]. Three previous analyses of QIAreach QFT have reported a high sensitivity and specificity relative to QFT-Plus [17,55,56], but the performance of the test has not previously been studied in children under 5, or in samples of population-representative individuals in a setting with high TB transmission [57]. A limitation of the study is that we are unable to also evaluate novel skin tests, due to the logistical demands of introducing a second visit for all participants [18], but we will save additional blood samples from participants which may allow future evaluation of other tests of Mtb infection.

Vertically-funded single-disease programmes are expensive and challenging to sustain. TB surveillance should optimally be integrated within existing systems, such as demographic health surveys, surveillance for other infections [37,58,59], or routine primary care–for example when children attend for vaccination visits. We have therefore chosen to evaluate recruitment in primary health centres, taking advantage of the high uptake of interventions such as childhood vaccinations, and Blantyre’s well-functioning primary healthcare system. A similar approach which screened clinic attendees for malaria parasitaemia was able to successful identify malaria “hotspot” villages in Chikwawa District, Malawi [58]. However, while recruitment in health centres is an attractive option with respect to feasibility, we recognise that children attending health centres may differ systematically from those in the population. We intend to address this potential limitation by 1) only recruiting children attending, or accompanying family members, on “routine” preventive health visits such as vaccinations or family planning; 2) performing convenience sampling in parallel with more conventional community-based sampling, and comparing the results, with respect both to the characteristics of the participants recruited and the estimates of Mtb infection prevalence.

Despite the widespread historic use of tuberculin surveys, there is very little qualitative research that has explored the acceptability of population-level surveillance for Mtb infection [6]. There is also little context-specific evidence surrounding the relative acceptability of blood- vs injection-based tests for Mtb infection, despite sensitivities around such tests in Malawi [60–63]. We have designed the study with the input of the MLW TB/HIV Community Scientific Advisory Board (a panel of volunteer community members affected by TB and HIV), and will include questionnaire items around testing acceptability, and a qualitative component, to explore the acceptability of this approach.

## Conclusion

As TB epidemiology changes, there is an urgent need for reactive, localised, high-quality sub-district epidemiological data to guide geographically targeted interventions and support modelling of interventions to high-risk groups. This study aims to explore the feasibility, acceptability and potential utility of surveillance based on Mtb infection in a well-characterised Malawian city with high HIV and TB prevalence and successful scale-up of HIV and TB interventions, which have led to recent rapid declines in both undiagnosed HIV and TB, and in TB case-notification rates. We will explore new and potentially sustainable clinic-based approaches that could contribute to countries such as Malawi meeting United Nations country commitments for TB preventive treatment, and support efficient targeting of interventions as the country moves towards TB elimination.
